# Bacterial and Archaeal Diversity in the Gastrointestinal Tract of the North American Beaver (*Castor canadensis*)

**DOI:** 10.1371/journal.pone.0156457

**Published:** 2016-05-26

**Authors:** Robert J. Gruninger, Tim A. McAllister, Robert J. Forster

**Affiliations:** Lethbridge Research and Innovation Centre, Agriculture and Agri-Food Canada, Lethbridge, Alberta, Canada; Wageningen University, NETHERLANDS

## Abstract

The North American Beaver (*Castor canadensis*) is the second largest living rodent and an iconic symbol of Canada. The beaver is a semi-aquatic browser whose diet consists of lignocellulose from a variety of plants. The beaver is a hindgut fermenter and has an enlarged ceacum that houses a complex microbiome. There have been few studies examining the microbial diversity in gastrointestinal tract of hindgut fermenting herbivores. To examine the bacterial and archaeal communities inhabiting the gastrointestinal tract of the beaver, the microbiome of the ceacum and feaces was examined using culture-independent methods. DNA from the microbial community of the ceacum and feaces of 4 adult beavers was extracted, and the16S rRNA gene was sequenced using either bacterial or archaeal specific primers. A total of 1447 and 1435 unique bacterial OTUs were sequenced from the ceacum and feaces, respectively. On average, the majority of OTUs within the ceacum were classified as Bacteroidetes (49.2%) and Firmicutes (47.6%). The feaces was also dominated by OTUs from Bacteroidetes (36.8%) and Firmicutes (58.9%). The composition of bacterial community was not significantly different among animals. The composition of the ceacal and feacal microbiome differed, but this difference is due to changes in the abundance of closely related OTUs, not because of major differences in the taxonomic composition of the communities. Within these communities, known degraders of lignocellulose were identified. In contrast, to the bacterial microbiome, the archaeal community was dominated by a single species of methanogen, *Methanosphaera stadtmanae*. The data presented here provide the first insight into the microbial community within the hindgut of the beaver.

## Introduction

Lignocellulose, the primary component of the plant cell wall, is a promising renewable resource for the production of a number of high-value products including bio-fuel, fine chemicals, livestock feed, and as low cost substrate for microbial fermentation and production of enzymes [1,2,3]. Efficient deconstruction of lignocellulose into fermentable sugars requires the concerted action of cellulases, hemicellulases and enzymes that hydrolyze the cross-linkages between hemicellulose and lignin [1,2].

In herbivores, the complete hydrolysis of these requires the synergistic activity of a wide range of carbohydrate degrading enzymes that are expressed by the microbes in their gastrointestinal (GI) tract [4]. The ecology of the gut microbiome in herbivores (ruminants, macropods, and hind-gut fermenters) has been the target of intensive research efforts [5,6,7,8,9]. The recently published Global rumen census has revealed that the microbiome composition of ruminants varies both as a function of diet and ruminant species [9]. Additionally, a number of studies have revealed differences in the microbiome of wild as compared to domesticated herbivores [5,7,8,10,11,12]. This has been hypothesized to be due to the varied diets that are available to wild animals [8]. The diets of domesticated herbivores usually consist of high-quality forages or concentrates (*e*.*g*. hays, silages, or grain concentrates), whereas the diets of wild herbivores are more varied, depending on the nature of the browse and forage available for consumption at a given point in time. Given the different feeding strategies that are utilized by wild and domesticated animals, one would expect that the microbial populations in these hosts should be distinct.

Rodents are primarily herbivores and utilize hind-gut fermentation to break down cellulosic feeds [13]. With the exception of the mouse (*Mus musculus*) and the lab rat (*Rattus norvegicus*), which serve as model systems for understanding the interactions between host and gut microbiome [14,15], few studies have examined the microbial communities in the ceacum of rodents [16,17]. Of particular interest, the microbial basis of lignocellulose digestion in one of the most iconic Canadian rodents, the beaver (genus *Castor*), has not been reported.

The North American beaver, *Castor canadensis*, is the second largest member of the rodent family and exhibits a semi-aquatic lifestyle [18]. Beavers can significantly impact an ecosystem by altering the water flow of streams and/or rivers. Beaver foraging of local vegetation is also known to have a considerable impact on the ecological succession, composition and structure of plant communities, making them an important keystone species in riparian ecosystems [18]. The beaver’s diet consists of woody, lignified plant material, bark, roots and aquatic plants. Beaver’s are hindgut fermenters possessing an enlarged ceacum where digestion and fermentation of the lignocellulosic material takes place [19,20]. Evidence of cellulase activity within the ceacal contents has previously been reported but the source of this activity has not been determined [19]. Undoubtedly, the ability of the beaver to breakdown lignocellulose can be attributed to the microbiome found in the GI tract, but the makeup of this community has not been examined.

This study aims to address this question by determining the bacterial and archaeal communities in the ceacum and feaces of the *Castor canadensis*. Illumina sequencing of the 16S rRNA gene using bacterial, and archaeal specific primers was used to determine the composition of the bacterial and archaeal microbiome in each sample. The ceacum and fecal microbiome of 4 beavers (2 males and 2 females) was examined and the variation in community structure between individuals and between the GI tract regions was undertaken.

## Materials and Methods

### Sample Collection

Samples were obtained from a licensed hunter during regular hunting activities and did not require any special permits. Animals were sacrificed by a single gunshot to the head, and no animals were sacrificed specifically for this study. All of the animals lived in the same area of southern Alberta in Lethbridge County. All animals were mature adults of uncertain age weighing approximately 18–22 kg and appeared to be of good health and were dissected within one hour of death. Approximately 5 g samples were taken from the ceacum. Fresh feacal pellets were collected from the lower colon. Samples were transported to the lab, frozen on liquid nitrogen and stored at -80°C.

### DNA Extraction

Frozen samples were ground in a Retsch RM 100 Mortar Grinder (Retsch, Newtown, PA) with the addition of 30 mL of 100mM Tris-HCl pH 8.0, 500mM EDTA pH 8.0, 1.5M NaCl, 1mg/mL Proteinase K in the presence of liquid nitrogen for 5 min. Following grinding, the samples were incubated at 50°C for 40 min, combined with 3 mL 2% SDS and incubated at 65°C for another 45 min. The lysate was centrifuged at 19,200 × g for 10 min at room temperature to pellet debris. The lysate supernatant was combined 1:1 (v/v) with 65°C 2% agarose, then poured into 90 mm square petri plates. The agarose containing the embedded DNA was equilibrated 3 times over 24 h against 30 volumes of TE (10 mM Tris pH 8.0, 1 mM EDTA pH 8.0) buffer and subsequently stored at 4°C. Large molecular weight DNA was eluted from the agarose using the “Freeze squeeze” method [21]. The DNA concentration was determined with a nanodrop (Thermo Fischer Scientific, Wilmington, DE) using the Quant-iT PicoGreen dsDNA assay kit according to the manufacturer’s protocol (Life Technologies, Burlington, ON).

### Illumina Sequencing

The 16S rRNA gene V1-V3 variable region PCR primers 27F 5’-AGAGTTTGATCMTGGCTCAG-3’ [22] and 519R 5’-AGRGTTTGATCMTGGCTCAG-3’ [23] were used in a 30 cycle PCR using the HotStarTaq Plus Master Mix Kit (Qiagen, Toronto, ON) under the following conditions: 94°C for 3 min, followed by 28 cycles of 94°C for 30 sec, 53°C for 40 sec and 72°C for 1 min, after which a final elongation step at 72°C for 5 min was performed. A barcode was included on the forward primer to allow for multiplexing of samples. After amplification, PCR products were checked in a 2% agarose gel to determine the success of amplification and the relative intensity of bands. Amplified products from the 4 ceacal and 4 feacal samples were pooled in equal proportions based on their molecular weight and DNA concentrations. Pooled samples were then purified using calibrated Ampure XP beads (Beckman Coulter, Mississauga, ON). The pooled and purified PCR product was used to prepare DNA libraries following the Illumina TruSeq DNA library preparation protocol. Sequencing was performed at MR DNA (www.mrdnalab.com, Shallowater, TX) on an Illumina MiSeq following the manufacturer’s guidelines. Archaeal diversity was assessed using the same sequencing protocol with the primers 349F 5’-GYGCASCAGKCGMGAAW-3’ and 806R 5’-GGACTACVSGGGTATCTAAT-3’ [24].

### Sequence Analysis

QIIME 1.9 was used for sequence analysis, OTU detection, taxonomic assignment and phylogenetic analysis [25]. Assembled reads were demultiplexed, and primers (both forward and reverse), short sequences (<350 bp) and sequences longer than 600 bp were removed. Sequences with >6 ambiguous base calls, homopolymeric runs exceeding 6 bp or with quality scores <25, and with errors in the primer or barcode were also removed. The remaining high quality sequences were clustered into OTUs at 94% ID for both bacterial and archaeal diversity. OTU clustering and detection/removal of chimeric sequences was carried out using USEARCH61 [26] using an open reference OTU picking approach. Rare, low abundance OTUs (<10 sequences) were not considered and were removed from the OTU table. Taxonomy was assigned based on the greengenes 13.8 reference sequence dataset [27] using UCLUST [26]. OTUs were aligned using PyNAST [28] and based on the aligned greengenes 13.8 reference dataset [27]. Aligned OTUs were used to construct a phylogenetic tree using FastTree [29]. The sequences for the bacterial and archaeal diversity have been deposited to the Small Reads Archive (NCBI) with accession numbers SRP069014 and SRP069012.

### Examination of Alpha- and Beta-Diversity

Microbial diversity within (Alpha-diversity) and between samples (Beta-diversity) was assessed using Qiime. Alpha-diversity measures for richness (Chao1), phylogenetic diversity (branch length based diversity), number of observed OTUs, and taxonomic abundance were evaluated. Sequences were subsampled to the lowest number of sequences found in all samples to ensure alpha and beta-diversity analysis used the same number of sequences per sample. Beta-diversity analysis was carried out using both quantitative and qualitative measures. Quantitative measures included weighted UniFrac and abundance based Jaccard coefficients which consider both phylogenic relationships and abundance of OTUs [30]. Qualitative measures included unweighted UniFrac and a binary Jaccard dissimilarity coefficient that only considers differences based on phylogeny [30]. Principle coordinate analysis of UniFrac distances and Jaccard coefficients was carried out to generate PcoA plots showing both quantitative and qualitative differences in community structure.

### Statistical Analysis

Scripts within Qiime were used to determine the statistical significance of comparisons. The Qiime script compare_categories using an ANOSIM test was used to examine whether there were statistically significant differences in samples based on location (ceacum vs feces), sex (male versus female), or Individual. To determine whether any OTUs were differentially abundant between location within the GI tract the script group_signficance was run using a Mann-Whitney U test. Only location within the GI tract was considered in this comparison. Comparison of alpha-diversity and beta-diversity metrics were done using the scripts compare_alpha_diversity and beta_diversity, respectively. Default parameters were used for these comparisons. P-values < 0.05 where designated as statistically significant.

## Results

### Analysis of Sequencing Data and Depth

Illumina sequencing of all 8 samples resulted in a total of 670,888 assembled reads. The reads were demultiplexed based on barcodes and extracted using the split_libraries.py command in Qiime. Only assembled reads with a quality score >25, a quality window of 25, no mismatches in the primer or barcode, homopolymers < 6 and within 350–600 bp in size were retained for further analysis. A total of 353,729 high quality reads were retained after filtering and utilized in the Qiime analysis of bacterial diversity in the beaver GI tract. The number of reads in the samples ranged from 50,937 (Male 2 feaces) to 33,993 (Female 2 feaces). OTU clustering resulted in a total of 1,675 unique OTUs that were identified across all 8 samples. The number of reads, number of unique reads and number of observed OTU's (94% identity cutoff) and measures of richness in each sample are shown in Table 1. Bacterial and Archaeal sequences were randomly subsampled to select 33,993 and 29,383 reads respectively, from all samples for use in downstream analysis of alpha- and beta-diversity.

**Table 1:** Summary of 16S rRNA gene sequencing of beaver ceacum and feacal samples for bacterial and archaeal diversity analysis.

Sample	Location	Reads (Total)	Reads (Unique)	Observed OTUs*	Choa1*	Phylogenetic Diversity*
*Bacterial Diversity*						
Male 1	ceacum	46139	1531	1465	1614	38.1
Male 1	feaces	39284	1440	1446	1590	37.8
Male 2	ceacum	40887	1471	1450	1628	37.7
Male 2	feaces	50937	1518	1489	1614	38.2
Female 1	ceacum	41073	1506	1446	1598	37.3
Female 1	feaces	52784	1549	1400	1634	36.2
Female 2	ceacum	48632	1539	1413	1619	36.9
Female 2	feaces	33993	1489	1417	1631	37.0
*Archaeal Diversity*						
Male 1	ceacum	168809	183	151	196	4.88
Male 1	feaces	78041	161	131	174	4.14
Male 2	ceacum	66616	118	101	131	3.63
Male 2	feaces	72143	135	113	151	4.26
Female 1	ceacum	97907	159	132	185	4.59
Female 1	feaces	76270	193	172	229	5.11
Female 2	ceacum	29361	107	100	118	3.59
Female 2	feaces	70642	187	181	213	5.15

*Bacterial diversity comparisons were carried out after randomly subsampling 33993 sequences to ensure an equal number of reads were used in all subsequent analysis. Archaeal sequences were similarly subsampled to 29361.

### Bacterial Community Composition in the Ceacum and Feaces of Beaver

**Fig 1:**
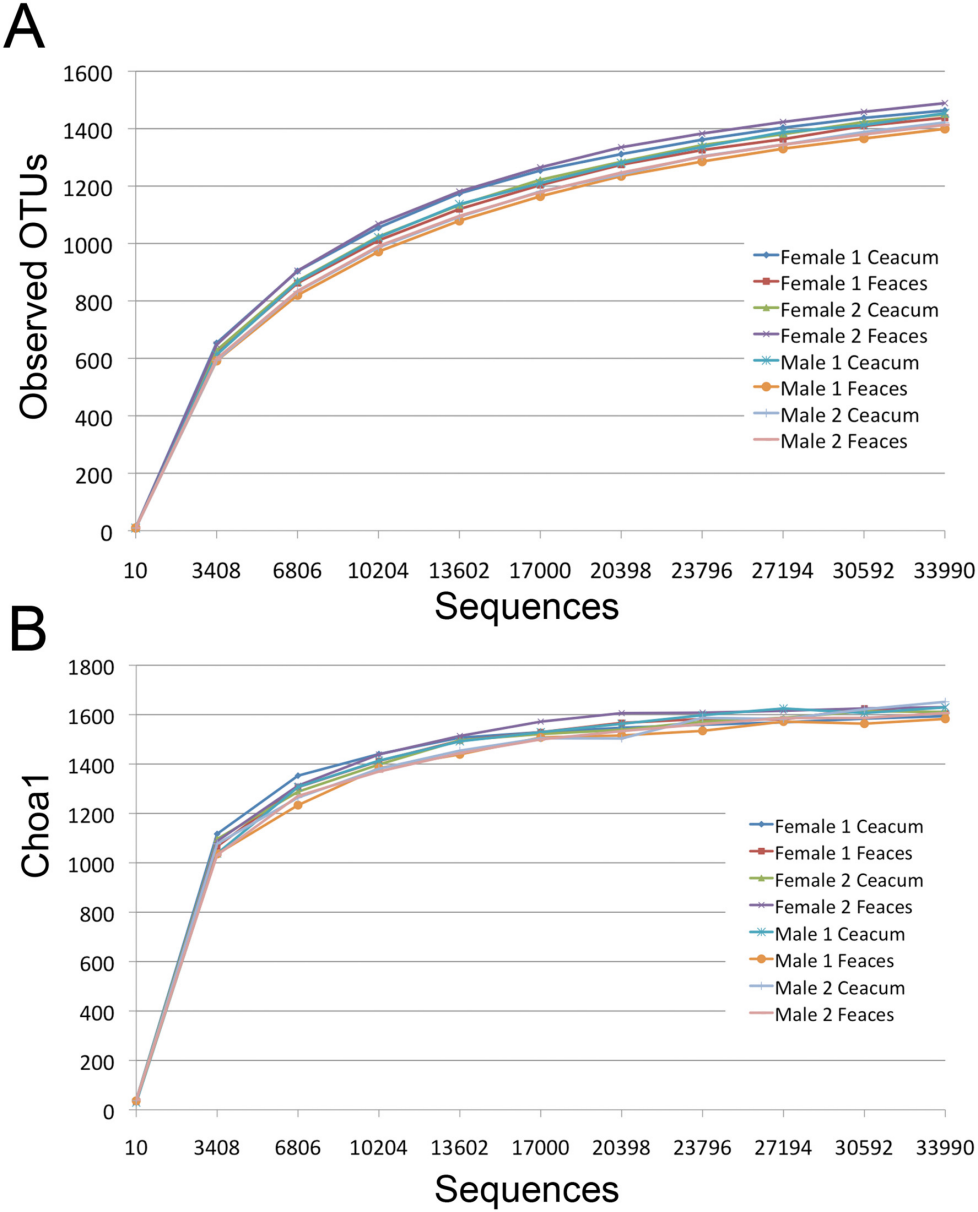
Rarefaction curves showing the A) Observed OTUs, B) Choa1 as a function of sequencing effort. Sequences from each sample were randomly sub-sampled to the lowest number of sequences contained in all samples (33993 sequences).

A number of alpha-diversity metrics were used to assess the similarity in the community structure in all of the beaver samples examined in this study. Phylogenetic diversity, Chao1 and the number of observed OTUs were used to quantify the community structure of the samples. Rarefaction curves were constructed to determine if the depth of sequencing was adequate to capture the full microbial diversity within samples. Rarefaction analysis showed that all samples reached a plateau at a similar level indicating that sequencing depth was sufficient to capture most of the microbial diversity in the samples (Fig 1). The number of observed OTUs in the ceacum and feaces was not statistically different (p value = 0.6). There was also no significant difference in the number of OTUs found among individuals or between sex. The phylogenetic diversity of the ceacum and feaces were 37.5 ± 0.5 and 37.3 ± 0.8. There was also no statistical difference in the species richness as measured by both Chao1 (1621 ± 22 for ceacum and 1606 ± 19 for feaces) and phylogenetic diversity (37.5 ± 0.5 for ceacum and 37.3 ± 0.8 for feaces) in samples from the ceacum versus feaces. Similarly, the number of observed OTUs based on rarefaction analysis revealed no difference in the number of OTUs in the ceacum (1447 ± 16) and feaces (1435 ± 34; Table 1).

**Fig 2:**
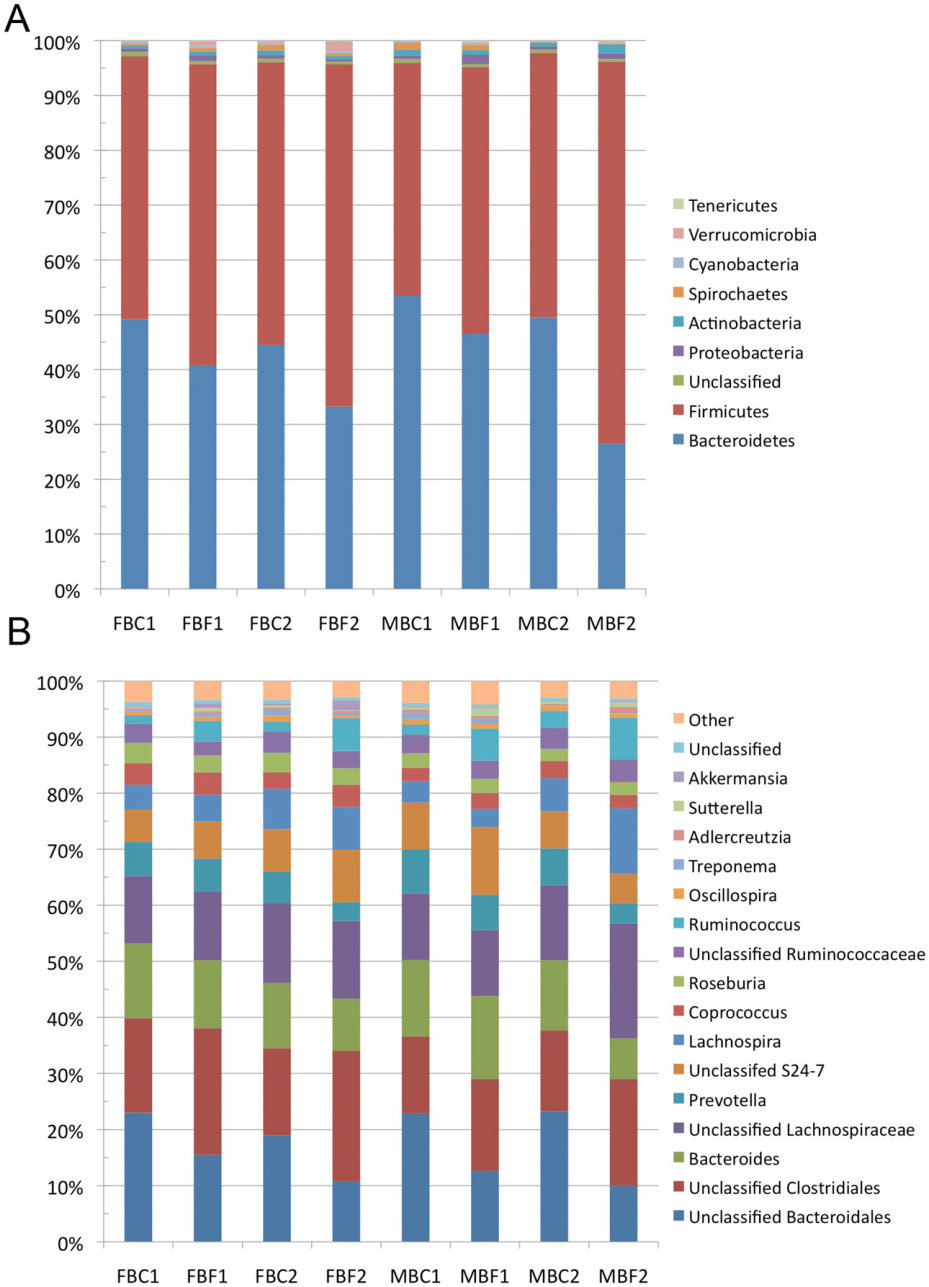
Taxonomic summary of 16S rRNA gene sequences from the ceacum and feaces of each beaver examined in this study. A) Relative abundance of sequences in the ceacum and feaces of each individual at the phylum level. B) Relative abundance of sequences in the ceacum and feaces of each individual at the genus level. Sequences present with < 0.5% relative abundance were pooled into a group labeled “other”.

The ceacum and fecal microbiomes were both predominated by Bacteroidetes and Firmicutes (Fig 2A). This was consistent in all of the animals examined in this study (Fig 2A). Although these phyla dominate in both locations of the GI tract, the relative abundance of the two phyla differed significantly in each location of the gut. Bacteroidetes was found in greater abundance in the ceacum as compared to the feaces (49.2% ± 3.7% versus 36.8% ± 8.7%, p-value = 0.04). Conversely, Firmicutes was less abundant in the ceacum as compared to the feaces (47.6% ± 3.7%, versus 58.9% ± 9.1%, (p-value = 0.06). Less abundant phyla identified in both the ceacum and feaces included Proteobacteria, Actinobacteria, Spirochaetes, Cyanobacteria, Verrucomicrobia, and Tenericutes (Fig 2A). The only low abundance phylum to show significant differences between ceacum and feaces was Tenericutes (0.04% ± 0.02% in ceacum; 0.16% ± 0.04% in feaces, p-value = 0.004). Classification at the phylum level was generally high, with only 0.68% of the OTUs in the ceacum, and 0.52% of the OTUs in the feaces not being classified to this taxonomic rank.

**Fig 3:**
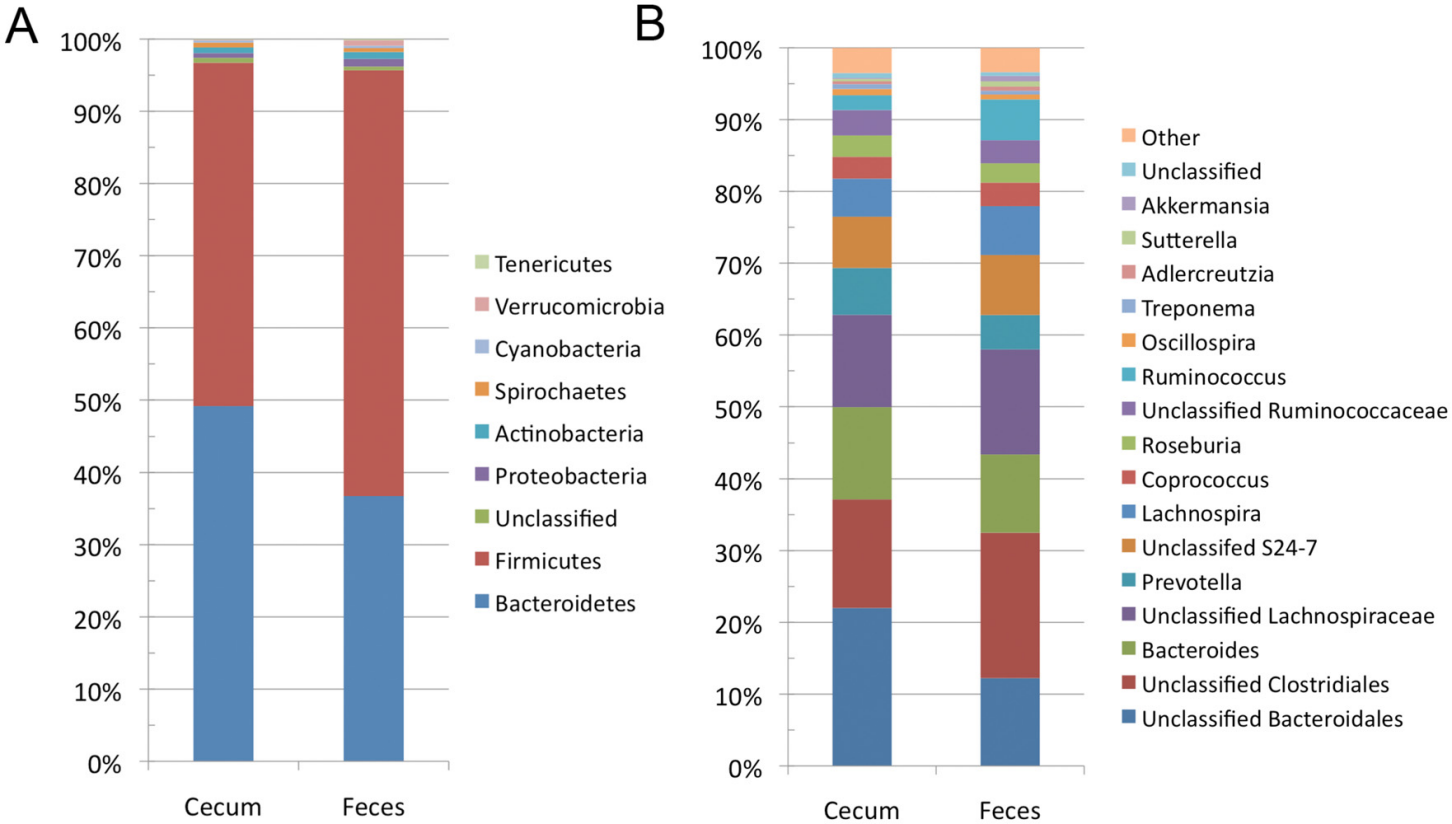
Average taxonomic summary of the bacteria identified in the ceacum and feaces of beavers at both the phylum and genus level. A) Average relative abundance of sequences in all ceacum and feaces samples at the phylum level. B) Average relative abundance of sequences in all ceacum and feaces samples at the genus level. Sequences present with < 0.5% relative abundance were pooled into a group labeled “other”.

Lachnospiraceae (25.4% in ceacum and 28.3% in feaces) was the most abundant family observed in both caecum and rectal samples. OTUs in the family Lachnospiraceae were assigned to an unidentified genus of Lachnospiraceae, *Lachnospira*, *Coprococcus* and *Roseburia* (Fig 2B). The most abundant OTUs in both the ceacum and feaces belonged to Unclassified Bacteroidales and Unclassified Clostridiales (Fig 3B). Other abundant OTUs were assigned to family Bacteroidaceae, Prevotellaceae, Ruminococcaceae, and S24-7. These OTUs were found in amounts ranging from 3.4% to 14.9%. The most abundant OTUs that could be classified to the genus level, were *Bacteroides* (7.3–14.8%), *Prevotella* (3.4–7.8%), *Lachnospira* (3.2–11.7%), *Coprococcus* (2.4–4.0%), *Roseburia* (2.2–3.7%), and *Ruminococcus* (1.5–7.5%). OTUs belonging to the genera: *Oscillospira*, *Treponema*, *Blautia*, *Clostridium*, *Dorea*, *Adlercreutzia*, *Sutterella*, *Akermansia*, and *Parabacteroides* were also observed at an abundance of > 0.1% in all samples.

### Differential Abundance of Microbial Community Members in Ceacum and Feaces

The taxonomic differences observed for the samples from the ceacum and feaces were further investigated to identify the OTUs that were differentially located between these sites. A total of 66 OTUs were differentially distributed between these two locations in the GI tract. Of these, 39 OTUs were more prevalent in the ceacum, and 27 were found at higher levels in the feaces. The 13 OTUs within the phylum Bacteroidetes and 22 OTUs within the phylum Firmicutes were preferentially located in the caecum. 4 OTUs could not be classified at any taxonomic level. Of the 22 OTUs within the phylum Firmicutes, 8 could only be classified to the order Clostridiales, 7 were classified as members of the family Ruminococcaceae, 7 were classified as members of the family Lachnospiraceae. The Bacteroidetes OTUs were all classified within the order Bacteriodales and included 9 OTUS that could not be classified further, 1 OTU belonged to the family Porphyromonadaceae, and 3 OTUs were classified to the genus Bacteroides. The OTUs that were more abundant in the feaces where primarily Firmicutes (25 out of 27). 16 of these OTUs could only be classified to the order level, and belonged to Clostridiales. 5 OTUs were classified in the family Ruminococcaceae, 3 OTUs were classified in the family Clostridiaceae. An OTU within the family Streptococcaceae was also found preferentially in the feaces. The 2 OTUs that were not in the phylum Firmicutes were classified in the phylum Bacteroidetes,and Verrucomicrobia.

### The Bacterial Community Composition of the Feaces and Ceacum Differ due to Changes in the Abundance of Microbial Taxa

**Fig 4:**
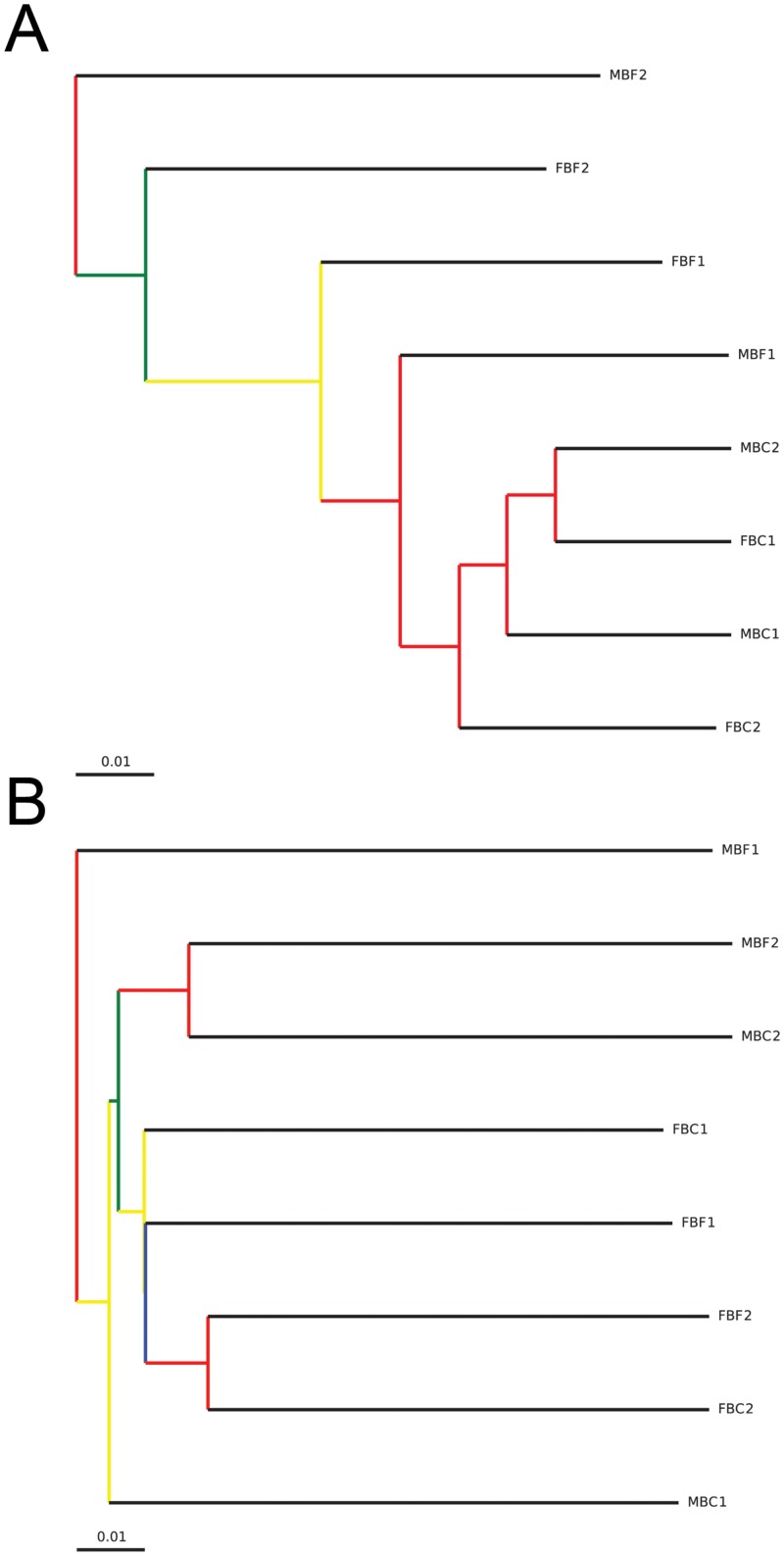
Hierarchical clustering of microbiomes from ceacum and feaces of 4 beavers. Distance matrices were generated with A) weighted and B) unweighted UniFrac calculations. Jack knife analysis was used to evaluate the reliability of the clustering results at even sequencing depth. Nodes colored red show 75–100% support, yellow 50–75%, green 25–50% and blue <25% support.

The Bray-Curtis dissimilarity metric and weighted UniFrac were both used to examine differences, or similarities in the microbial community with consideration for both the occurrence and abundance of OTUs. Unwieghted UniFrac calculations were carried out to examine differences between communities based solely on the occurrence of OTUs. Comparison of the ceacum and feaces microbiome based on the Bray-Curtis dissimilarity metric showed that these communities differed in both composition and abundance of OTUs within each community (P-value = 0.001). Clustering of samples based on a weighted UniFrac analysis showed that the ceacum samples clustered separately from the fecal samples (P-value = 0.023) (Fig 4A). The branch points on the tree of the weighted UniFrac clustering are supported by high bootstrap values (> 0.75). Clustering of samples based on an unweighted UniFrac analysis revealed that samples from Female beaver 1 and 2, and Male beaver 2 clustered according to individual (P-value = 0.01) (Fig 4B). Bootstrap values for clustering of Male beaver 2 and Female beaver 2 samples were high (> 0.75) but those of Male and Female 1 clusters were less reliable.

**Fig 5:**
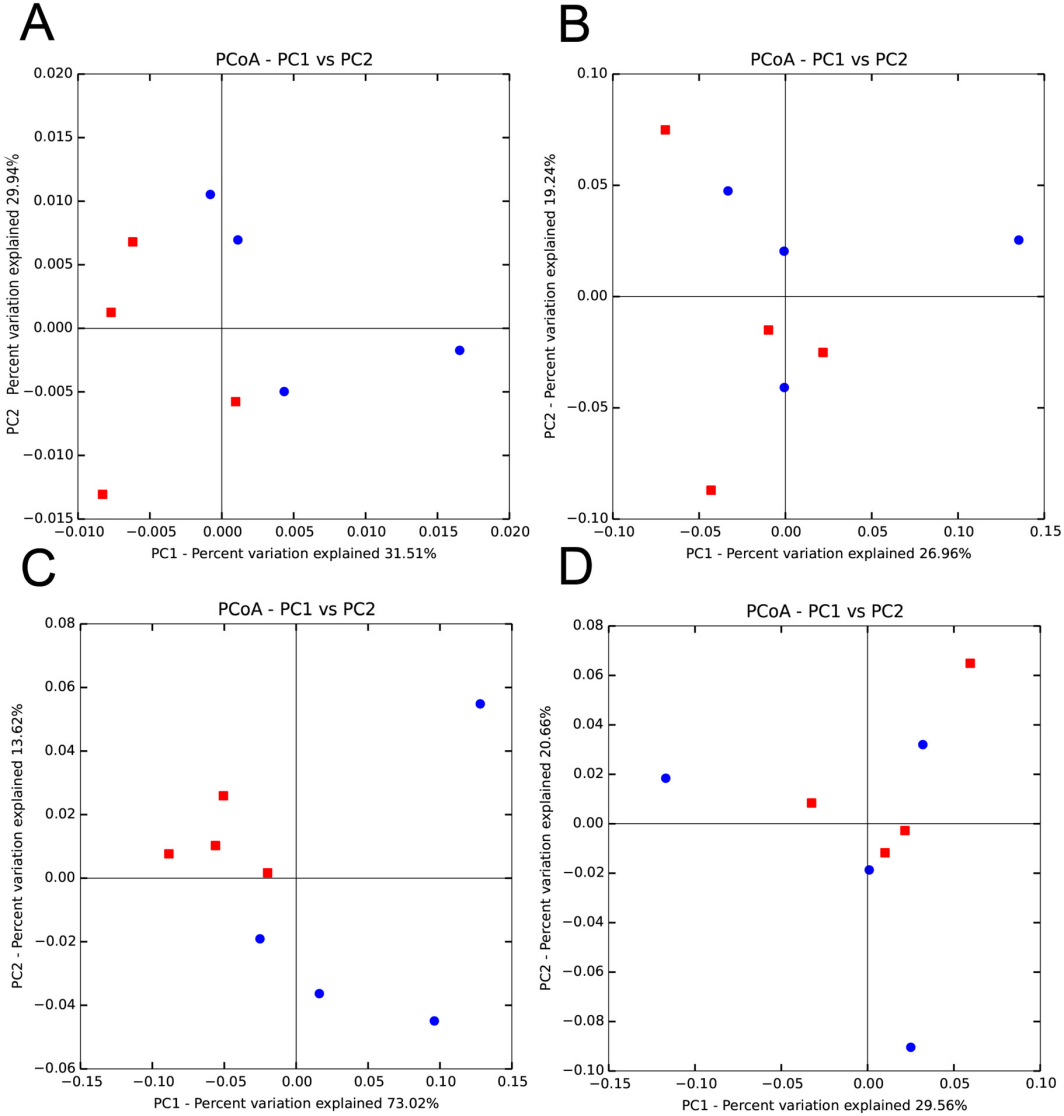
Principle coordinate analysis (PcoA) of the ceacum (red squares) and feacal (blue circles) microbiomes. Comparisons were carried out using both qualitative and quantitative measures of alpha and beta diversity. A) PcoA using abundance based Jaccard dissimilarity coefficient, B) PcoA using binary Jaccard dissimilarity coeffiecient, C) PcoA based on weighted UniFrac, D) PcoA based on unweighted UniFrac. The percent variation explained by each principle coordinate is shown.

The differences in the communities of the ceacum and feaces could also be observed in principle coordinate analysis plots using distance matrices from both Jaccard and UniFrac analysis. A principle coordinate plot of distances calculated using a Jaccard analysis that considers OTU abundance, showed clustering of the ceacum samples, whereas the fecal samples exhibited a more disperse distribution (Fig 5A). In contrast, a plot of distances calculated using a binary Jaccard dissimilarity coefficient, which only considers qualitative differences, showed that the ceacum and fecal samples were both diffusely distributed and did not cluster separately (Fig 5B). Similarly, a principal coordinate plot of distances calculated using a weighted UniFrac showed clear separation of the ceacum and feacal communities (Fig 5C), however samples did not cluster separately in a plot based on unweighted UniFrac distances (Fig 5D).

### Archaeal Community Composition in Ceacum and Feaces of Beaver

To determine the composition of the archaeal community in the samples, the archaeal selective 16S rRNA gene primer set 349F/806R was used [24]. Illumina sequencing of the 8 samples resulted in a total of 877,115 assembled reads. The same quality control parameters that were employed for the bacterial specific primer set were applied to the archaeal sequences. After demultiplexing and quality control 660,984 sequences were retained for analysis with Qiime. There was a much wider variation in the number of archaeal sequences in each sample as compared to the bacterial sequences (Table 1). The sample from the ceacum of the Male beaver 1 had by far the highest number of sequences (169,144) whereas the sample from the ceacum of the Female beaver 2 had the least (29,383). Sequences were randomly subsampled to select 29,383 reads from all samples to use in the comparative analysis of the archaeal communities. Clustering of sequences from all samples at 94% sequence identity resulted in 208 unique OTUs.

In contrast to the high bacterial diversity found in the ceacum and feaces of beavers, only 3 genera of archaea were found, *Methanosphaera*, *Methanobrevibacter*, and *Thermoplasmatales*. After removal of any OTUs that were classified to the kingdom Prokaryote there were 10 Archaeal OTUs. 5 of these OTUs classified as *Methanosphaera*, 4 OTUs as *Methanobrevibacter* and 1 as *Thermoplasmatales*. In all samples, *Methanosphaera* was the predominant archaea detected with >99% of the archaeal reads being assigned to this species in all but one sample. A single OTU accounted for 85–90% of the *Methanosphaera* sequences. A search of the non-redundant nucleotide database in NCBI using blastn identified this OTU as *Methanosphaera stadtmanae* DSM 3091. Clustering sequences into OTUs at 94% identity level may result in multiple species within a genus to cluster together so it is possible that there are more than one species of *Methanosphaera* within this OTU. *Methanobrevibacter* was observed at low levels in 6 of the 8 samples ranging from 0.02–3.3% of the archaeal reads. The OTUs classified to this genus were all classified as “uncultured Archaea” or “uncultured *Methanobrevibacter* sp.”. *Thermoplasmatales* was observed in one of the fecal samples with an abundance of 0.47% and was identified as an uncultured, unknown species. There was no significant difference in the species richness, phylogenetic diversity or number of observed OTUs between locations in the GI tract, or among individuals.

## Discussion

The diet of the beaver consists primarily of lignocellulosic material and this mammal has evolved to efficiently utilize this abundant feedstuff [18]. The aim of this study was to examine the microbial community in the hindgut of the beaver and examine the extent with which the community varies between sites within the GI tract, and among individuals. The data presented also provides insight into how similar or different the gut microbiome of the beaver is to other herbivores.

Herbivores have evolved 3 distinct mechanisms to digest the plant cell wall; foregut (ruminants), macropod foregut, and hindgut fermentation [4,6,7]. Although the physiological basis of digestion is unique, all three approaches utilize a highly specialized microbiome that is adapted to deconstructing lignocellulose. Early research into lignocellulose digestion in the beaver GI tract identified cellulase activity in ceacal contents [19], although the microbial basis of this activity was not investigated. Our results demonstrate that the microbial community found in the ceacum and feaces of the beaver is similar to that found in other hindgut fermenters [6,16,17,31]. These bacterial communities in the beaver are both dominated by Firmicutes and Bacteroidetes; a characteristic that is common within all mammalian guts [6]. It was interesting that in all of the samples examined >95% of the OTUs belong to these two phyla. This indicates that the microbial community in the beaver shows less taxonomic diversity than the “typical” mammalian gut characterized by Ley et al (2008). In the largest scale study of the mammalian gut microbiome to date, 17 phyla were detected with the most abundant (on average) being: Firmicutes (65.7%), Bacteroidetes (16.3%), Proteobacteria (8.8%), Actinobacteria (4.7%) and Verrucomicrobiota (2.2%), with other phyla present at <1%. Network analysis identified both species and diet as primary determinants of microbiome composition. Interestingly, herbivores can be differentiated based on fermentation mechanism [6]. Subsequent work has probed more deeply into the microbiome of a number of herbivores. The economic importance of ruminants has led to a great deal of effort into understanding the rumen microbiome. Most recently, the Global Rumen Census provided a global view of the core rumen microbiome [9] with 67.1% of all sequence data, in all 742 samples being *Prevotella*, *Butyrivibrio*, *Ruminococcus*, unclassified *Lachnospiraceae*, *Ruminococcaceae*, *Bacteroidales* and *Clostridiales*. All members of this core microbiome fall within the phyla Bacteroidetes (*Prevotella*, *Bacteroidales*), or Firmicutes (*Butyrivibrio*, *Ruminococcus*, *Lachnospiraceae*, *Clostridiales*). We could not identify a core microbiome in the samples we examined. Unlike the rumen, *Butyrivibrio* and *Fibrobacter* were not found in the beaver hindgut. *Fibrobacter* was found in 93% of all rumen samples examined, although the prevalence and abundance of this genus varied in different ruminant species and as a function of diet [9]. *Fibrobacter* is found in the gut microbiota of many herbivores and plays an important role in lignocellulose digestion in these organisms [9,32].

Although lacking, there have been some studies carried out on herbivorous rodents. The composition of the gut microbiome in the wood rat (*Neotoma* sp) over the entire GI tract has been determined [16]. In contrast to the beaver, the ceacum of the wood rate is dominated by Firmicutes whereas the dominant phyla in the feces is Bacteroidetes. The bacterial community in the ceacum of the capybara has been characterized using a PhyloChip. The most prevalent phyla found in these samples were Firmicutes (34.5%), Proteobacteria (32.3%), Bacteroides (8.1%), Actinobacteria (7.4%) and Spirochetes (4.0%). The high prevalence of Proteobacteria compared to Bacteroides is not typical of the gut bacterial community but is likely due to inherent biases in the probes present on the PhyloChip. A study using this same approach to examine the rumen microbiome in Moose using both PhyloChip also showed unusually high levels of Proteobacteria [33]. When next-generation sequencing was used to examine the bacterial community in the Moose rumen, Bacteroidetes and Firmicutes were found to be the most abundant bacterial phyla [11].

Macropods employ a foregut based mechanism to digest plant material a physiology that differs from ruminants [7]. The most abundant phyla (>1% abundance) in the foregut of 3 species of Grey Kangaroo were Bacteroidetes (48.3%), Firmicutes (47.3%), Proteobacteria (1%) and Fibrobacteres (1%) with over 90% of the OTUs belonging to Bacteroidetes and Firmicutes [7]. Similarly, over 95% of the OTUs from both the ceacum and feaces of beaver were Bacteroidetes and Firmicutes.

Mammals are not the only vertebrates that have evolved to digest plant cell walls. Several studies have also been carried out on the microbial community in the hindgut of herbivorous reptiles and a unique folivorous bird, the Hoatzin, that possesses an enlarged crop where fermentation takes place [34,35,36,37]. The bacterial community in the hindgut of herbivorous reptiles is dominated by Firmicutes and Bacteroidetes and shows similarities to that found in hindgut fermenting mammals. Lignocellulose digestion occurs in the enlarged crop of the Hoatzin. UniFrac community analysis places the microbial community in the Hoatzin as more similar to that found in the rumen than to other birds [36]. This indicated that the primary factor determining microbial community is location of fermentation (foregut versus hindgut) [36], a conclusion the supports the findings of Ley et al [6].

There were no significant differences in the microbial communities in the ceacum or feaces of the beavers examined in this study. However, a comparison of the phylogenetic composition of the ceacal and feacal microbiomes revealed that these communities are distinct (Figs 2 & 3). This difference is only apparent when both OTU abundance and taxonomy are considered (Figs 4 & 5). Our results indicate that the composition of the feacal and ceacal microbiome differ, but that this difference is a result of changes in the abundance of closely related OTUs as opposed to significant differences in the taxonomical composition of the communities. The difference in the communities is not entirely unexpected given the distinct physiological processes that take place in these sites with regard to the fermentation of lignocellulose within the ceacum.

A large percentage of the OTUs making up the community were uncharacterized. The presence of a large number of uncharacterized bacteria in gut environments is commonly observed [6,9,16,17,37]. The bacterial community in the crop of the Hoatzin also had a large number of OTUs (94%) that were unclassified at the species level [37]. The Global Rumen Census found that 70% of the identified OTUs were not classifiable to the genus level [9]. Most of the uncharacterized/classified OTUs in the GI tract of the beaver are members of the Clostridiales and Bacteroidales families. Within these families there are a number of celluloytic bacteria [38,39,40]. A BLASTn search of 16S rRNA gene sequences from several unclassifiable OTUs using the non-redundant NCBI database with default search parameters, identified that many of the these OTUs have similarities to uncultured gut microbes, including a number of uncultured rumen bacteria. This may indicate that some of the mechanisms used in ruminants for deconstructing the plant cell wall are shared by the microbiome in the beaver GI tract. Further studies are needed to confirm this.

We did identify microbes in the GI tract of the beaver that are known to produce carbohydrate active enzymes including: *Prevotella*, *Clostridium*, and *Rumminococcus*. *Rumminococcus* was found at high levels (3.2–6.2%) in all samples and many of these OTUs were similar to *R*. *flavefaciens*; a species of bacteria known to be involved in lignocellulose degradation in the rumen [41]. OTUs similar to *R*. *flavefaciens* where found to be more prevalent in feaces than the ceacum. The feaces of the beaver consisted primarily of coarse chunks of undigested wood. *R*. *flavefaciens* expresses cellulosomes that facilitate adhesion of the microbe to plant cell walls [42] and the high levels of undigested lignocellulose in the feaces may provide a niche for these bacteria to occupy.

Ongoing research into the gut microbiome and its role in metabolism continues to uncover novel mechanisms of cellulose digestion that are utilized by previously uncharacterized bacteria [43]. Uncultured and uncharacterized lineages of Bacteroidetes are found in all ecosystems specializing in lignocellulose degradation [38,39]. It had been thought that Bacteroidetes where only able to metabolize soluble polysaccharides and did not play a direct role in the saccharification of crystalline cellulose. However, a recent study of uncharacterized Bacteroidetes identified a novel polysaccharide utilization loci based mechanism of cellulose degradation not known to exist in this class of bacteria [38,39]. In a separate study, a novel cellulolytic fibre degrading bacterium related to the rumen bacteria, *R*. *flavifaciens* was isolated from human feaces and found to share orthologous gene clusters involved in degrading crystalline cellulose [44]. Recent studies into the role that uncharacterized bacteria in the gut play in the digestion of cellulose leads to the hypothesis that some of the uncharacterized bacteria in the ceacum and feaces of the beaver are involved in the digestion of lignocellulose. It is important to note that the function of a microbiome is not only related to its composition, but also to the genes that are present, and being actively transcribed. Future studies examining the genomic composition or transcriptional activity could foster information on the molecular mechanisms of cell wall deconstruction utilized by the gut microbiome of the beaver.

In addition to examining the prokaryotic diversity, we examined the archaeal community in the beaver GI tract. Unlike the bacterial microbial community, the composition of the archaeal community was very limited with >99% of the archaeal sequences corresponding to *Methanosphaera stadtmanae*. *M*. *stadtmanae* is dependent on acetate as a carbon source and generates methane through the reduction of methanol with H_2_ [45]. This result was unexpected because the archaeal community in the gut of herbivores is generally dominated by *Methanobrevibactor* species [9,46]. However *Methanosphaera stadtmanae* was found to be the predominant methanogen in the kangaroo forestomach [47], although it was present in quite low numbers. *Methanosphaera stadtmanae* was also found to be the predominant phylotype in the GI tract of Sumatran Orangutans [48]. This was hypothesized to be due to the high levels of pectin in the leaves and fruit that make up this animals diet, resulting in high levels of methanol being produced as a result of pectin metabolism [48]. Another study examining the archaeal diversity in the folivorous howler monkeys found similar levels of *M*. *stadtmanae* [49]. Folivores have been identified as having unique gut microbiomes in comparison to other herbivores [6]. This may indicate that pectin is an important energy source for the beaver and that acetate is an abundant VFA generated in the beaver hind-gut. The low abundance and diversity of methanogens in the GI tract of the beaver may also explain the observation that these rodents produce very low levels of methane [50]. This may also suggest that the main pathway for hydrogen removal in the beaver gut is through reductive acetogenesis. Reductive acetogenesis has been shown to be the main pathway for hydrogen removal in the forestomach of kangaroo [51]. It was hypothesized that the acetogenic bacteria *Blautia* spp. is primarily responsible for reductive acetogenesis in the kangaroo forestomach [51]. *Blautia* sp. was found in all samples so it is possible that a similar method of hydrogen disposal occurs in the hind-gut of beavers.

### Conclusion

This study sheds light on the makeup of the microbiome in beaver. The composition of the bacterial microbiome consists primarily of Firmicutes and Bacteroidetes and a number of OTUs within these phyla have low sequence identity to characterized phylotypes. The composition of the feaces and ceacum microbiome is different, but this difference is due to changes in the abundance of closely related OTUs, not from significant differences in the taxonomic composition of the communities. The archaeal community in both the feaces and ceacum was dominated by a single methanogen, *M*. *stadtmanae*, suggesting that acetate is an important energy source in the beaver and that methane is generated through the reduction of methanol. Within the beaver gastrointestinal tract, several known degraders of lignocellulose were identified. There large number of Unclassified Bacteroidales and Unclassified Clostridiales make it probable that novel, uncharacterized bacteria with plant cell wall degrading activity play a role in digestion in the beaver GI tract. Further studies that focus on the metabolic activity of these communities and the enzymes they express are needed to provide further details on the enzymatic process that is utilized by the beaver microbiome to facilitate the digestion of lignocellulose.
